# Phosphorylated CPI-17 and MLC2 as Biomarkers of Coronary Artery Spasm–Induced Sudden Cardiac Death

**DOI:** 10.3390/ijms25052941

**Published:** 2024-03-03

**Authors:** Yiming Dong, Jianfeng Wang, Chenteng Yang, Junxia Bao, Xia Liu, Hao Chen, Xiaojing Zhang, Weibo Shi, Lihua Zhang, Qian Qi, Yingmin Li, Songjun Wang, Rufei Ma, Bin Cong, Guozhong Zhang

**Affiliations:** 1 Hebei Key Laboratory of Forensic Medicine, Collaborative Innovation Center of Forensic Medical Molecular Identification, College of Forensic Medicine, Hebei Medical University, Shijiazhuang 050017, China; 23031100043@stu.hebmu.edu.cn (Y.D.); 20212152@stu.hebmu.edu.cn (J.W.); chn_yct4750@hebmu.edu.cn (C.Y.); 102023011@btmc.edu.cn (J.B.); 17800586@hebmu.edu.cn (X.L.); 22033100283@stu.hebmu.edu.cn (H.C.); 17800745@hebmu.edu.cn (X.Z.); 18401452@hebmu.edu.cn (W.S.); 20201058@stu.hebmu.edu.cn (L.Z.); 18300784@hebmu.edu.cn (Q.Q.); 16000557@hebmu.edu.cn (Y.L.); 17800584@hebmu.edu.cn (S.W.); 18200713@hebmu.edu.cn (R.M.); cong6406@hebmu.edu.cn (B.C.); 2 Hebei Province Laboratory of Experimental Animal, Shijiazhuang 050017, China

**Keywords:** coronary artery spasm, PKC, CPI-17/MLC2 signaling, biomarker

## Abstract

Coronary artery spasm (CAS) plays an important role in the pathogeneses of various ischemic heart diseases and has gradually become a common cause of life-threatening arrhythmia. The specific molecular mechanism of CAS has not been fully elucidated, nor are there any specific diagnostic markers for the condition. Therefore, this study aimed to examine the specific molecular mechanism underlying CAS, and screen for potential diagnostic markers. To this end, we successfully constructed a rat CAS model and achieved in vitro culture of a human coronary–artery smooth-muscle cell (hCASMC) contraction model. Possible molecular mechanisms by which protein kinase C (PKC) regulated CAS through the C kinase-potentiated protein phosphatase 1 inhibitor of 17 kDa (CPI-17)/myosin II regulatory light chain (MLC2) pathway were studied in vivo and in vitro to screen for potential molecular markers of CAS. We performed hematoxylin and eosin staining, myocardial zymogram, and transmission electron microscopy to determine myocardial and coronary artery injury in CAS rats. Then, using immunohistochemical staining, immunofluorescence staining, and Western blotting, we further demonstrated a potential molecular mechanism by which PKC regulated CAS via the CPI-17/MLC2 pathway. The results showed that membrane translocation of PKCα occurred in the coronary arteries of CAS rats. CPI-17/MLC2 signaling was observably activated in coronary arteries undergoing CAS. In addition, in vitro treatment of hCASMCs with angiotensin II (Ang II) increased PKCα membrane translocation while consistently activating CPI-17/MLC2 signaling. Conversely, GF-109203X and calphostin C, specific inhibitors of PKC, inactivated CPI-17/MLC2 signaling. We also collected the coronary artery tissues from deceased subjects suspected to have died of CAS and measured their levels of phosphorylated CPI-17 (p–CPI-17) and MLC2 (p-MLC2). Immunohistochemical staining was positive for p–CPI-17 and p-MLC2 in the tissues of these subjects. These findings suggest that PKCα induced CAS through the CPI-17/MLC2 pathway; therefore, p–CPI-17 and p-MLC2 could be used as potential markers for CAS. Our data provide novel evidence that therapeutic strategies against PKC or CPI-17/MLC2 signaling might be promising in the treatment of CAS.

## 1. Introduction

Coronary artery spasm (CAS) refers to local or diffuse reversible excessive contractions in coronary arteries due to various stimuli, causing partial or complete occlusion of the artery under either normal or atherosclerotic conditions [1]. CAS plays an important role in the pathogeneses of various types of ischemic heart diseases such as angina, acute myocardial infarction (MI), and sudden cardiac death (SCD) [2,3]. Forensic pathologists often encounter cases of sudden death in which the deceased had experienced conflicts with others or mild trauma. Some such cases are found to have no significant lethal disease or injury on autopsy, as well as no specific pathomorphological diagnostic markers, and cause of death is determined to be “unknown” or “sudden cardiac death”. In forensic practice, coronary artery disease (CAD) is the leading cause of SCD, and the main cause of CAD is coronary atherosclerosis or CAS [4]. If the deceased had significant predisposing factors (e.g., disputes with others, overexertion, cold stimulus), it should be highly suspected that CAS was the cause of death [1]. However, potential biomarkers for diagnosis of CAS-induced SCD remain largely unknown. Researchers must reveal the molecular mechanisms underlying the lethality of CAS, and the markers of this condition, to provide a forensic reference for suspected SCD, new directions for clinical treatment, and a reduction in the mortality rate.

Studies have shown that hypercontractility of vascular smooth muscle (VSM) is an important mechanism that induces CAS [5]. Smooth-muscle contraction is mainly dependent on myosin II regulatory light chain (MLC2), the phosphorylation level of which is affected by the ratio of myosin light chain kinase (MLCK) activity to myosin light chain phosphatase (MLCP) activity. Studies have found that MLC2 phosphorylation is an important process in CAS [6]. Abnormal endoplasmic reticulum (ER) stress can induce CAS by regulating the MLCK/MLC2 pathway [7]. The above-cited studies suggest that MLC2 phosphorylation might be key to CAS onset. Smooth-muscle cells (SMCs) maintain their contractile function through calcium-sensitive mechanisms [8]. Kinase C-potentiated protein phosphatase 1 inhibitor of 17 kDa (CPI-17) is an important molecule that regulates calcium sensitivity. CPI-17 is associated with contraction of smooth muscles in different organs, such as those in the uterus during pregnancy, those in the bronchi in bronchial asthma, bladder detrusor muscles in type 1 diabetes (T1D) patients, and the mesenteric artery and aorta in db/db mice with type 2 diabetes (T2D) [9,10,11,12]. However, whether the CPI-17/MLC2 signaling pathway participates in coronary artery SMC contraction has not been reported.

Protein kinase C (PKC) is a serine/threonine kinase that is ubiquitous in almost all cell types and participates in many physiological and pathological processes. It is considered to comprise a family of many Ca^2+^-dependent and -independent isoforms with different enzymatic characteristics, substrates, and functions [13]. PKC regulates downstream signals such as endothelin-1 (ET-1), mitogen-activated protein kinase (MAPK), and CPI-17 to play an important role in vasoconstriction [14,15,16,17]. However, whether PKC also plays a role in regulating the CPI-17/MLC2 signaling pathway in CAS remains unclear.

The present study aimed to assess the potential of phosphorylated CPI-17 (p–CPI-17) and MLC2 (p-MLC2) for use as potential markers for CAS. To this end, we performed in vivo and in vitro experiments to investigate whether PKC regulated the CPI-17/MLC2 signaling pathway to mediate CAS. Coronary arteries from forensic cases were then assessed for the validation of candidate biomarkers. This allowed us to elucidate the potential molecular mechanism of PKC-induced CAS through the CPI-17/MLC2 pathway, and we conclude that p–CPI-17 and p-MLC2 could be used as potential markers for CAS.

## 2. Results

### 2.1. Myocardial and Coronary-Artery Injury in an Angiotensin II (Ang II)–Induced CAS Rat Model

**Figure 1:**
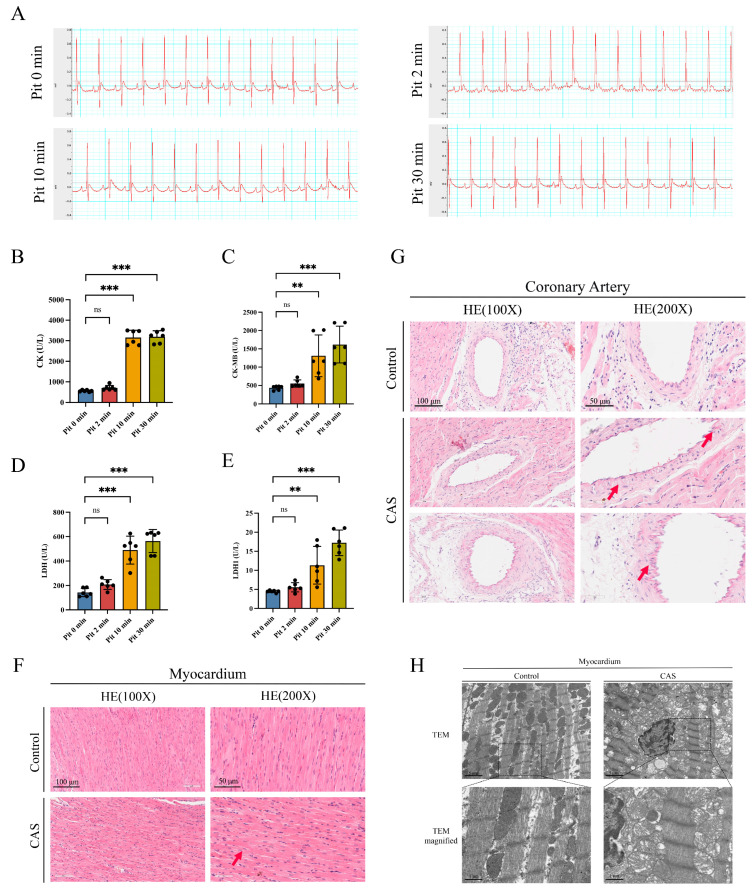
CAS caused myocardial injury and morphological changes to coronary artery tissues. (**A**) Representative ECG records of rats. (**B**–**E**) Serum levels of CK, CK-MB, LDH, and LDH1 at different time points after Pit injection in rats. *n* = 6 per group. (**F**) Representative H&E-stained images of rat myocardial tissues. Red arrow: swelling of myocardial fibers. Scale bar from left to right: 100 µm; 50 µm. (**G**) Representative H&E-stained images of rat LADAs. Red arrows: SMC vacuolation and increased endothelial and tunica intima folding. *n* = 6 per group. Scale bar from left to right: 100 µm; 50 µm. (**H**) Representative TEM images of rat myocardial tissues. Scale bar from left to right: 5 µm; 1 µm. Data are presented as mean ± standard error of the mean (SEM); ns, no significance; ** *p* < 0.01 and *** *p* < 0.001 vs. control group. Pit: pituitrin; CK: creatine kinase; CK-MB: creatine kinase isoenzyme MB; LDH: lactate dehydrogenase; LDH1: lactate dehydrogenase isoenzyme 1; CAS: coronary artery spasm; TEM: transmission electron microscopy.

In this study, we constructed a rat CAS model through tail vein injection of pituitrin (Pit) [7]. Electrocardiograms (EKGs), recorded at 0, 2, 10, and 30 min after injection, showed that the S-T segment was elevated in rats 2 min after Pit injection but had recovered to normalcy at 10 and 30 min after injection (Figure 1A). In addition, we measured creatine kinase (CK), creatine kinase isoenzyme MB (CK-MB), lactate dehydrogenase (LDH), and lactate dehydrogenase isoenzyme 1 (LDH1) levels in rat serum. Compared with the control group, there was no significant difference 2 min after Pit injection, but CK, CK-MB, LDH, and LDH1 expression were significantly increased at 10 and 30 min after injection (Figure 1B–E). Overall, the S-T segment elevation and increased myocardial enzyme levels we observed indicated successful construction of the CAS model.

To observe histomorphological changes in the rat myocardia and coronary arteries after CAS, we used hematoxylin and eosin (H&E) staining to analyze these tissues in both groups of rats. Our results showed that in CAS rats, myocardial fibers were swollen, spaces became narrow (Figure 1F), coronary artery SMC vacuolation occurred, and endothelial and tunica intima folding increased (Figure 1G). Additionally, internal elastic lamina and tunica intima folding significantly changed after CAS. These results were consistent with previously reported morphological post-CAS changes [18]. To obtain morphological ultrastructure evidence, we employed scanning electron microscopy (SEM). We found that in rats from the control group, myocardial fibers were mostly neatly arranged on both sides of myofilaments and of uniform size, with a tight myofilamental structure and satisfactory mitochondrial structure. However, in the CAS group, there was mild to moderate mitochondrial swelling, membrane rupture, matrix dissolution, crista rupture, shortening, and vacuolation (Figure 1H).

### 2.2. Treatment of Human Coronary Artery Smooth-Muscle Cells (hCASMCs) with Ang II–Promoted Contractile Activity In Vitro

**Figure 2:**
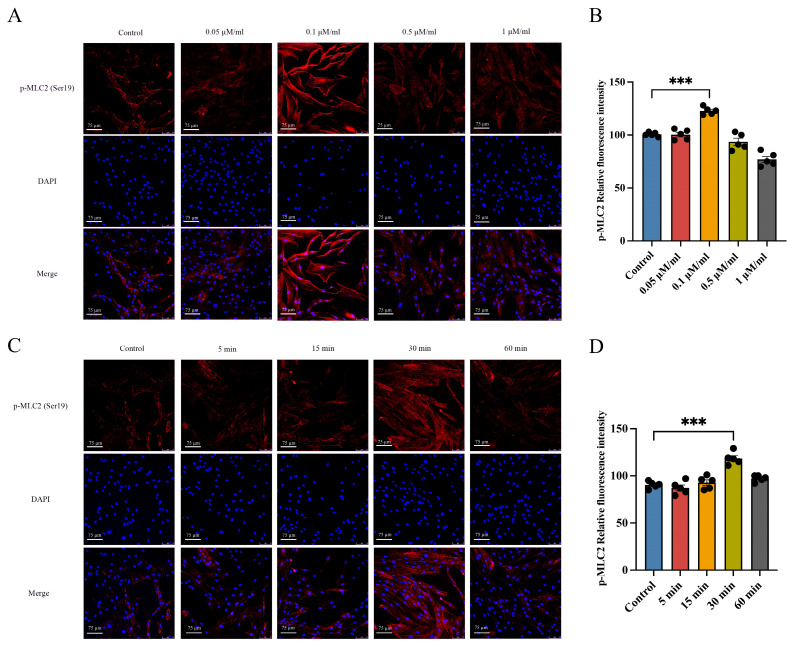
Treatment of hCASMCs with Ang II increased phosphorylation levels of MLC2 and promoted hCASMC contractile activities. We treated hCASMCs with different doses of Ang II. IF was used to measure the expression level of p-MLC2 (**A**,**B**) to observe cytoplasmic myoneme myofilament formation in hCASMCs. We exposed hCASMCs to constant doses of Ang II (0.1 μM/mL), which were administered based on increase in hCASMCs with time. IF was used to measure p-MLC2 expression level (**C**,**D**). *n* = 5 per group. Scale bar: 75 µm. Data presented as mean ± SEM. *** *p* < 0.001 vs. control group. hCASMCs: human coronary artery smooth-muscle cells; Ang II: angiotensin II; MLC2: myosin II regulatory light chain; p-MLC2: phosphorylated myosin II regulatory light chain.

Treatment with the vasoconstrictor Ang II is an effective method of simulating cell contraction in vitro [19]. Therefore, to study whether the PKC/CPI-17 signaling pathway participated in SMC contraction in vitro, we used Ang II to culture hCASMCs. Alpha-smooth-muscle actin (α-SMA) is a marker protein in SMCs. We first validated isolated hCASMCs via microscopy and α-SMA IF and found that the purity of hCASMCs was 90% (Figure S1). To select the optimal concentration and dosing time for hCASMC contraction, we added Ang II at different doses and time points into the culture medium. MLC2 is an important part of thick myofilament on which smooth-muscle contraction chiefly relies. Increased MLC2 phosphorylation indicates that hCASMCs have strong contractile activity. Therefore, using IF, we found that p-MLC2 levels in hCASMCs increased during Ang II treatment and peaked at a dose of 0.1 μM/mL before starting to decrease (Figure 2A,B). When we used a constant dose of 0.1 μM/mL Ang II to treat hCASMCs, we observed that MLC2 showed a bell-shaped phosphorylation curve and that significant phosphorylation peaked after 30 min (Figure 2C,D). These results show that Ang II induced hCASMC contraction and p-MLC2 phosphorylation in vitro in a dose- and time-dependent manner.

### 2.3. CPI-17 and MLC2 Were Hyper-Phosphorylated in the Rat CAS Model

**Figure 3:**
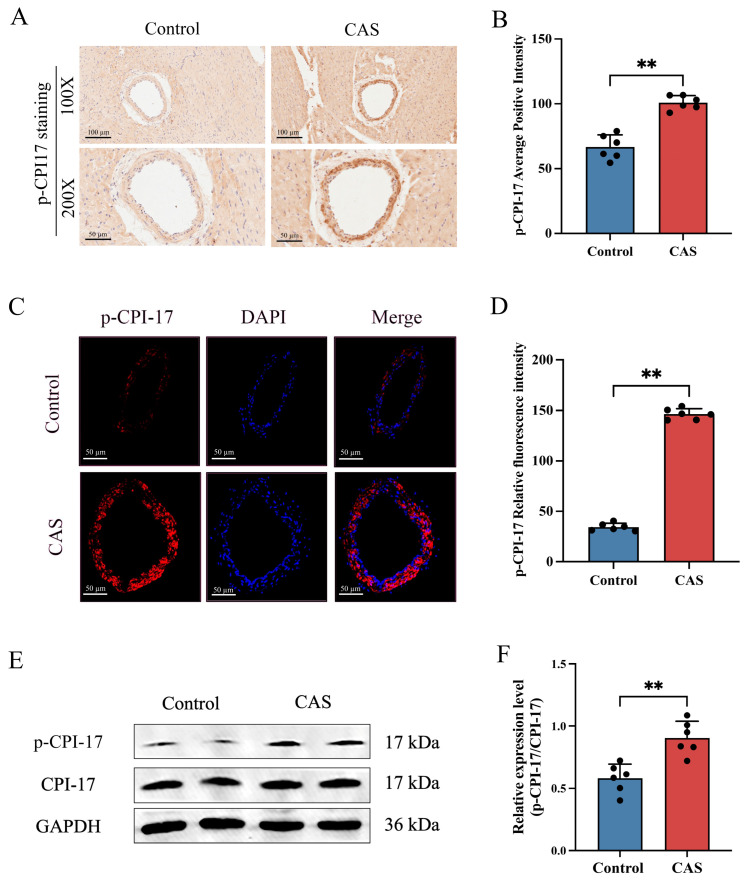
Effects of CAS on the expression of phosphorylated CPI-17 in rat coronary arteries. (**A**,**B**) Representative photomicrographs and semi-quantitative data of p-CPI-17 protein levels detected via IHC staining. Lower enlarged images are from upper images. *n* = 6 per group. Scale bar: 100 µm (upper images) or 50 µm (lower images). (**C**) Representative immunofluorescent images of p-CPI-17 (red) and DAPI (blue) in the coronary arteries of rats. Scale bar: 50 µm. (**D**) The mean fluorescence intensity of p-CPI-17 per section was quantified. *n* = 6 per group. (**E**,**F**) Western blot and quantitative analysis of p-CPI-17 protein levels of rat coronary arteries. *n* = 6 per group. Data presented as mean ± SEM. ** *p* < 0.01 vs. control group. CPI-17: C kinase-potentiated protein phosphatase 1 inhibitor of 17 kDa; p-CPI-17: phosphorylated C kinase-potentiated protein phosphatase 1 inhibitor of 17 kDa.

**Figure 4:**
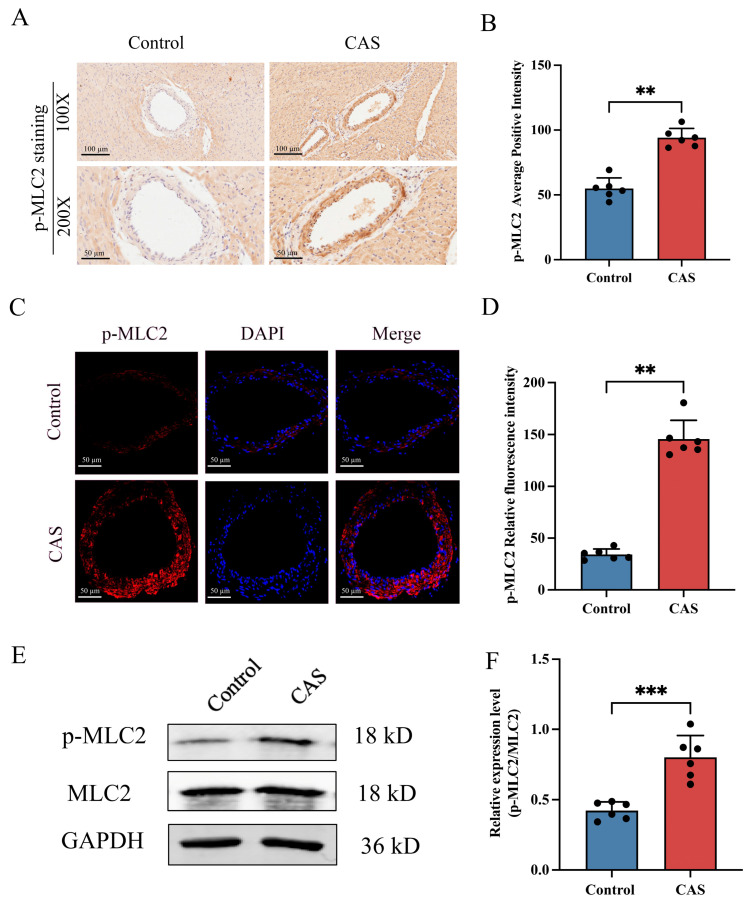
Effects of CAS on the expression of phosphorylated MLC2 in rat coronary arteries. (**A**,**B**) Representative photomicrographs and semi-quantitative data of p-MLC2 protein levels detected via IHC staining. Lower enlarged images are from upper images. *n* = 6 per group. Scale bar: 100 µm (upper images) or 50 µm (lower images). (**C**) Representative immunofluores-cent images of p-MLC2 (red) and DAPI (blue) in the coronary arteries of rats. Scale bar: 50 µm. (**D**) The mean fluorescence intensity of p-MLC2 per section was quantified. *n* = 6 per group. (**E**,**F**) Western blot and quantitative analysis of p-MLC2 protein levels of rat coronary arteries. *n* = 6 per group. Data presented as mean ± SEM. ** *p* < 0.01 and *** *p* < 0.001 vs. control group.

CPI-17 might be one of the important downstream factors that promote vasoconstriction [17,20]. Smooth-muscle contraction is mainly dependent on MLC2, the phosphorylation of which is an important process in CAS [6]. To analyze CPI-17 and MLC2 phosphorylation levels in rat coronary arteries after CAS, we used IHC and IF. The results showed that the coronary arteries of control group rats were negative for p–CPI-17. Meanwhile, those of rats from the CAS group were positive for p–CPI-17; the relevant staining was mainly concentrated in the tunica media (Figure 3A–D). Western blot (WB) analysis showed similar elevation trends in CPI-17 phosphorylation levels (Figure 3E,F) but no significant change in CPI-17 at the total protein level (Figure S2A). Similarly, MLC2 phosphorylation was significantly increased in rat coronary arteries after CAS (Figure 4A–D), but total MLC2 protein showed no significant change (Figure S2B). These results suggest that CAS stimulated the increases in p–CPI-17 and p-MLC2 in rat coronary arteries in vivo. 

### 2.4. PKC Translocation and Activity Were Increased in the Rat CAS Model and hCASMC Contraction Model

**Figure 5:**
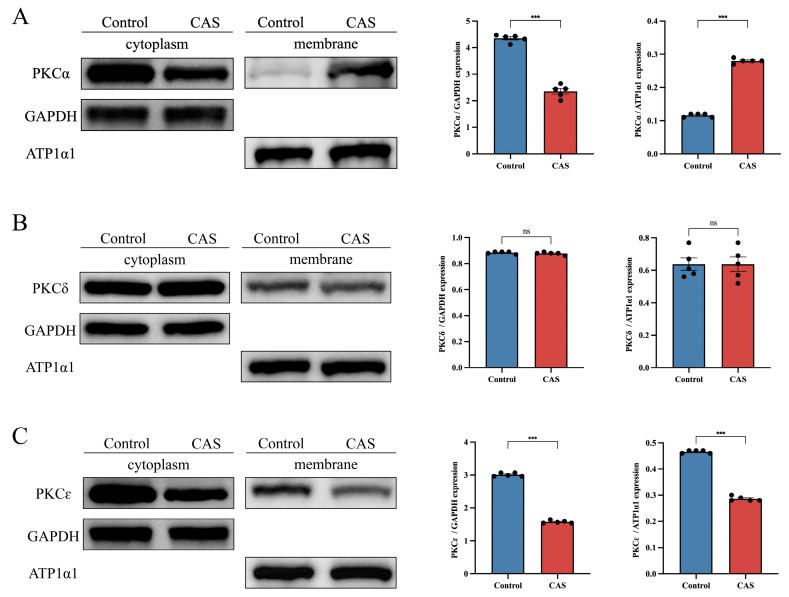
PKC was activated during contraction of CAS coronary arteries in vivo (*n* = 5 per group). Pit-induced PKC translocation in coronary arteries. (**A**) Representative WB shows PKCα in cytosolic and membrane particulate fractions. Results are expressed as the grayscale ratio of cytoplasmic PKCα to cell membrane PKCα. (**B**) Representative WB shows PKCδ in cytosolic and membrane particulate fractions. Results are expressed as the grayscale ratio of cytoplasmic PKCδ to cell membrane PKCδ. (**C**) Representative WB shows PKCε in cytosolic and membrane particulate fractions. Results are expressed as the grayscale ratio of cytoplasmic PKCε to cell membrane PKCε. Data presented as mean ± SEM. ns, no significance; *** *p* < 0.001 vs. control group. PKC: protein kinase C.

**Figure 6:**
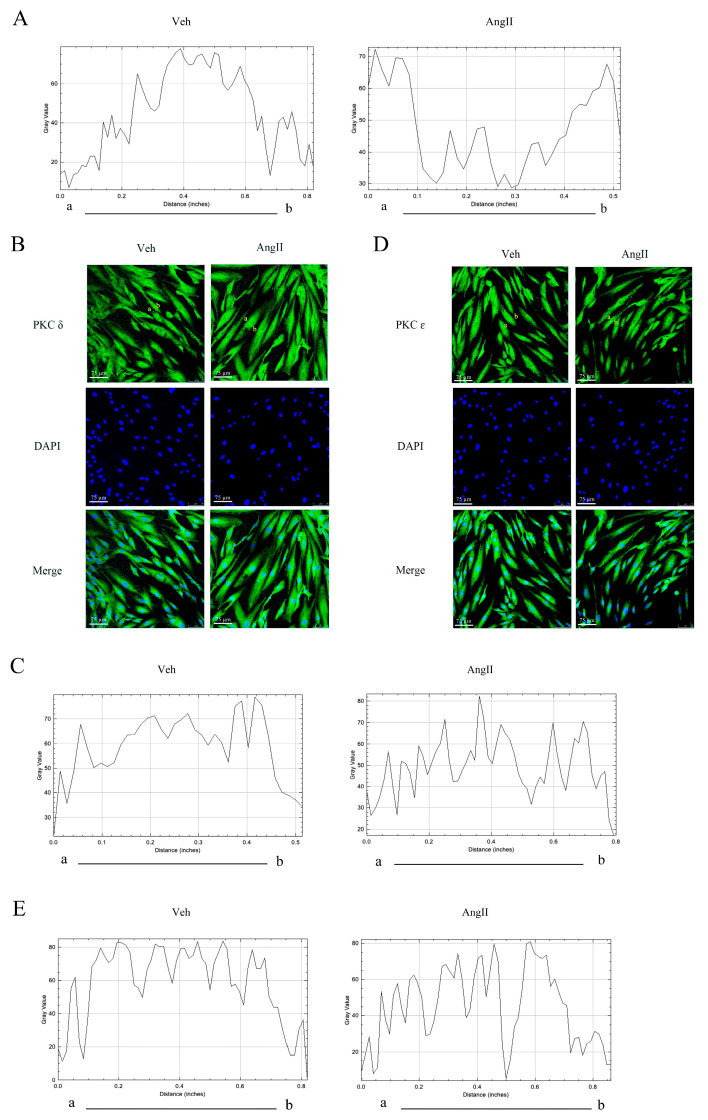
PKC was activated during contraction of hCASMCs in vitro (*n* = 5 per group). Ang II induced redistribution of PKC in hCASMCs. (**A**) The curve represents changes in PKCα fluorescence intensity along the “a” to “b” axis. The plot profile of the intensity from “a” to “b” was performed using ImageJ software (version 1.8.0; U.S. National Institutes of Health, Bethesda, MD, USA, https://imagej.net/ij/index.html, accessed on 18 June 2023). (**B**,**C**) Representative IF images of PKCδ (green) and DAPI (blue) in hCASMCs. Scale bar: 75 µm. The curve represents changes in intensity along the “a” to “b” axis. The plot profile of the intensity from “a” to “b” was performed using ImageJ software. (**D**,**E**) Representative IF images of PKCε (green) and DAPI (blue) in hCASMCs. Scale bar: 75 µm. The curve represents changes in intensity along the “a” to “b” axis. The plot profile of the intensity from “a” to “b” was performed using ImageJ software. Data presented as mean ± SEM.

**Figure 7:**
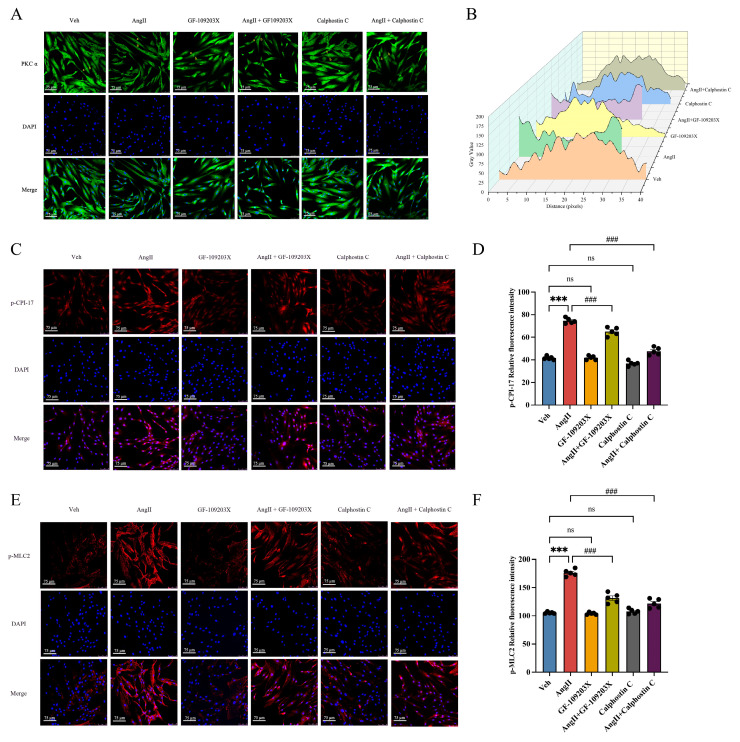
PKC mediates CPI-17/MLC2 signaling-induced contraction in cultured hCASMCs. (**A**) Representative immunofluorescent images of PKCα (green) and DAPI (blue) in hCASMCs. Scale bar: 75 µm. (**B**) The 3D waterfall plot results of PKCα immunofluorescence staining. The abscissa is the length of the cell section, and the ordinate is the gray value from a to b. (**C**) Representative immunofluorescent images of p-CPI-17 (red) and DAPI (blue) in cultured hCASMCs. Scale bar: 75 µm. (**D**) The mean fluorescence intensity of p-CPI-17 per section was quantified. *n* = 5 per group. (**E**) Representative immunofluorescent images of p-MLC2 (red) and DAPI (blue) in cultured hCASMCs. Scale bar: 75 µm. (**F**) The mean fluorescence intensity of p-MLC2 per section was quantified. *n* = 5 per group. Data presented as mean ± SEM. ns, no significance. *** *p* < 0.001 vs. control group; ^###^ *p* < 0.001 vs. Ang II group.

PKC plays an important role in regulating smooth-muscle contraction [21]. To pinpoint which PKC isoform participated in CAS, we tested the activity and protein expression of PKCα, PKCδ, and PKCε. WB revealed the presence of Ca^2+^-dependent PKCα and Ca^2+^-independent PKCδ and PKCε isoforms. In vehicle-treated rat coronary arteries, PKCα and PKCε were mainly distributed in cytoplasm, while PKCδ was evenly distributed between cytoplasm and the cell membrane. In the coronary arteries of CAS rats, cell membrane PKCα was increased while cytoplasmic PKCα was decreased (Figure 5A). Interestingly, we saw no significant changes in PKCδ or PKCε translocation in rat coronary arteries in the control group (Figure 5B,C). To validate the in vivo data, we exposed hCASMCs to 0.1 μM Ang II for 30 min and used IF to analyze the distribution changes of PKC isoforms. In resting cells, PKCα was mainly distributed in the cytoplasm, but Ang II induced its translocation to the cell membrane, which was accompanied by cell contraction (Figure 6A and Figure 7A). However, Ang II did not induce PKCδ or PKCε redistribution (Figure 6B–E). The above results show that PKCα translocation participated in CAS and cell contraction.

### 2.5. PKC Inactivation Decreased CPI-17/MLC2–Mediated hCASMC Contraction

CPI-17 is considered an endogenous mediator of the PKC signaling pathway [22,23]. We hypothesized that PKC played an important role in CAS induced by the CPI-17/MLC2 pathway. To study whether PKC could activate the CPI-17/MLC2 pathway to participate in CAS, we used GF-109203X and calphostin C (specific inhibitors of PKC) to treat hCASMCs. Next, we measured phosphorylation levels of CPI-17 and MLC2, critical proteins in the CPI-17/MLC2 pathway. As shown in Figure 7A,B, calphostin C treatment decreased Ang II-induced PKCα translocation in hCASMCs. Because GF-109203X and calphostin C treatment both decreased PKC activity in these cells, both drugs inhibited PKC signal transduction [24]. Subsequent IF analysis showed that p-CPI-17 and p-MLC2 showed significantly enhanced fluorescence intensity in hCASMCs after stimulation with 0.1 μM/mL Ang II for 30 min, but PKC inhibition decreased phosphorylation levels of CPI-17 and MLC2 (Figure 7C–F). Therefore, our study results support the hypothesis that PKCα activates the CPI-17/MLC2 pathway to induce cell contraction.

### 2.6. CPI-17 and MLC2 Phosphorylation Was Increased in the Coronary Arteries of Patients Suspected to Have Suffered CAS-Induced Sudden Cardiac Death

To further study the potential of p–CPI-17 and p-MLC2 as biomarkers for targeted forensic diagnosis of CAS in the near future, we evaluated the levels of both these proteins in human coronary arteries from autopsy cases. Forensic pathologists often see cases of sudden death after interpersonal conflicts or mild trauma, and some such cases show no signs of significant lethal disease or injury on autopsy. Note that physiological factors such as fatigue, mental stress, conflicts, and cold stimuli are factors that predispose patients to CAS [1]. We studied three cases in which the deceased had experienced conflicts and/or emotional agitation before death and no lethal pathological changes were found on autopsy, as suspected CAS-induced cardiac death cases; meanwhile, we also studied three non-cardiac death cases with definite causes of death as negative controls. All six cases are summarized in Supplementary Table S1. We selected p–CPI-17 and p-MLC2 as potential molecular markers for postmortem diagnosis of CAS in our Pit-induced CAS rat model and Ang II-induced hCASMC contraction model.

**Figure 8:**
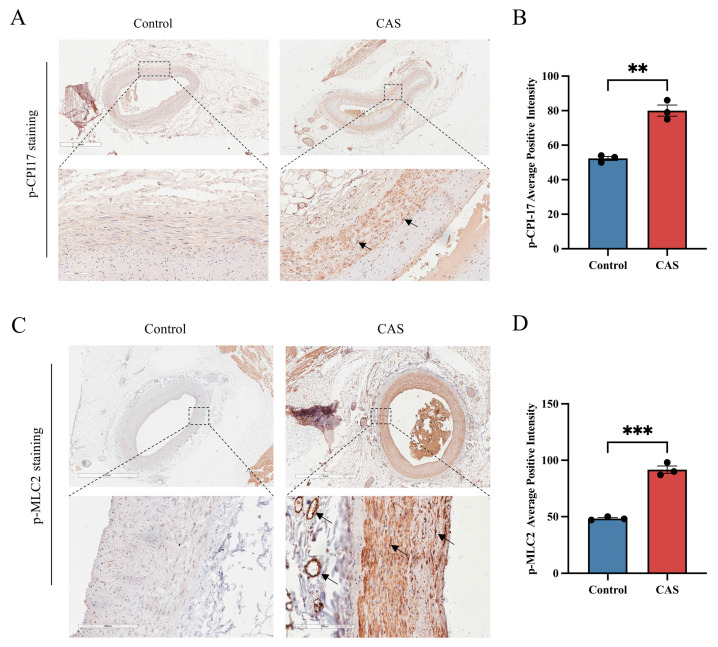
CPI-17 and MLC2 were moderately to highly phosphorylated in coronary arteries in deceased patients suspected to have suffered CAS-induced SCD (*n* = 3 per group). (**A**,**B**) Representative photomicrographs and semi-quantitative data of p–CPI-17 protein levels as detected by IHC staining. Bottom images are enlargements of top images. Arrows: positive staining for p–CPI-17 in vascular SMCs. Scale bar: 1 mm (top) or 100 µm (bottom). (**C**,**D**) Representative photomicrographs and semi-quantitative data of p-MLC2 protein levels detected via IHC staining. Bottom images are enlargements of top images. Arrows: positive staining for p-MLC2 in vascular SMCs. Scale bar: 2 mm (top) or 200 µm (bottom). Data presented as mean ± SEM. ** *p* < 0.01 and *** *p* < 0.001 vs. control group.

Interestingly, IHC produced positive p–CPI-17 staining in the left anterior descending artery (LADA) tissue of the suspected CAS-induced cardiac death cases, compared with equivalent tissues of the non-cardiac death cases (Figure 8A,B). In addition, in the suspected CAS-induced cardiac death cases, staining showed that coronary artery segments and neighboring interstitial arterioles were positive for p-MLC2 (Figure 8C,D). Overall, these data provide profound evidence that p–CPI-17 and p-MLC2 could be used as potential molecular markers for postmortem diagnosis of CAS.

## 3. Discussion

CAS is a pathological process of myocardial ischemia caused by transient hypercontraction of coronary arteries and characterized by sudden chest pain. It can cause myocardial ischemia, MI, and even sudden death [25]. CAS can significantly affect patient quality of life and prognosis, placing a huge economic burden on health services [26,27]. In forensic practice, CAS is highly suspected as the cause of death in many sudden-death cases if there are significant predisposing factors (e.g., disputes with other people, overexertion, cold stimulus) [28]. However, such cases lack specific diagnostic biomarkers; therefore, the identification opinions are controversial, resulting in adverse social effects. Elucidating the potential pathological molecular mechanisms of CAS is thus an urgent clinical and forensic practice issue that requires solutions.

One study [29] has reported that CAS presentation can be seen in early coronary angiography, but the limitations of this imaging modality in forensic pathology limit its use for diagnosing cause of death as CAS. Some researchers have explored specific markers of CAS. Factor et al. [30] were the first to find that smooth-muscle contraction bands are associated with CAS and that the degree of internal elastic lamina folding in CAS can reflect vasoconstriction and vasodilation status. These two findings could be used as morphological diagnostic characteristics of CAS. In our rat CAS model constructed through tail vein injection of Pit [9], results showed that myocardial fibers were swollen, spaces were narrow, coronary artery SMC vacuolation occurred, and endothelial and tunica intima folding increased. These results are consistent with previously reported morphological changes after CAS [18].

Coronary artery SMC hyperreactivity is a critical mechanism that causes CAS [31]. Smooth-muscle contraction efficiency is determined by the sensitivity of contractile proteins to calcium. CPI-17 is an important molecule that regulates calcium sensitivity. When CPI-17 is phosphorylated at threonine 32, it can specifically inhibit MLCP and increase MLC2 phosphorylation, in turn increasing actin–myosin cross-linking and thereby promoting polymerization of actin microfilaments and regulating SMC contraction. CPI-17, which is associated with smooth-muscle tonic contractions in various organs and participates in disease occurrence [9,10,11,12], was initially determined to be a substrate of PKC phosphorylation. Subsequent research found that Rho kinase, protein kinase N (PKN), and integrin-linked kinase (ILK) can also phosphorylate CPI-17. In addition, Rho kinase/receptor for activated C kinase 1 (RACK1) can exert its effects by promoting the PKC/CPI-17 phosphorylation pathway [32]. These findings indicate that CPI-17 might be a common and critical nexus of many intracellular kinases regulating MLCP and inducing CAS. Our results show that CPI-17 phosphorylation was significantly increased in coronary artery smooth muscle in CAS rats. In our in vitro experiments, consistent with our in vivo results, p–CPI-17 showed significantly enhanced fluorescence intensity in hCASMCs after 30 min of stimulation with 0.1 μM/mL Ang II. Taken together, all these results show that CPI-17 phosphorylation level participated in rat CAS and hCASMC contraction.

MLC2 is an important part of thick myofilament; smooth-muscle contraction mainly relies on it. MLC2 phosphorylation level is affected by the ratio of MLCK activity to MLCP activity. MLCP can remove high-energy phosphates from p-MCL2, dephosphorylating it and thereby inducing VSM relaxation. Studies have found that MLC2 phosphorylation is an important process in CAS [6] and that abnormal ER stress can induce CAS by regulating the MLCK/MLC2 pathway [6,7]. Our previous human tissue experiments showed that SMCs have high contraction reserve during mild coronary artery sclerosis and that MLC2 phosphorylation level and α-SMA expression are high in coronary artery smooth muscle. The above-cited results suggest that MLC2 phosphorylation might be key to CAS onset. Therefore, we measured the expression level of p-MLC2 in CAS rat coronary arteries and in the hCASMC contraction model. Our data initially showed elevated p-MLC2 levels in CAS rat coronary arteries stimulated with the vasoconstrictor Ang II, which also increased p-MLC2 levels in hCASMCs. These results indicate that MLC2 hyperphosphorylation accompanied vasoconstrictor-induced smooth-muscle contraction. We further evaluated the effects of MLC2 phosphorylation and dephosphorylation on hCASMC contraction by hypothesizing a causal relationship between an increase in p-MLC2 level and smooth-muscle contraction. MLC2 phosphorylation activates the intracellular cytoskeleton contraction–relaxation cycle and is favorable to contractile activity; MLC2 dephosphorylation reduces relaxation of coronary artery smooth muscle. We added Ang II into the culture medium at specific time points. Based on our observations, short-term exposure of hCASMCs to the vasoconstrictor caused phosphorylation of MLC2 and improved the arrangement of myofilaments. When exposure time was prolonged, MLC2 became dephosphorylated, resulting in disorderly myofilaments and decreased fluorescence. These observations support our hypothesis that MLC2 phosphorylation is a cause of hCASMC hypercontraction.

**Figure 9:**
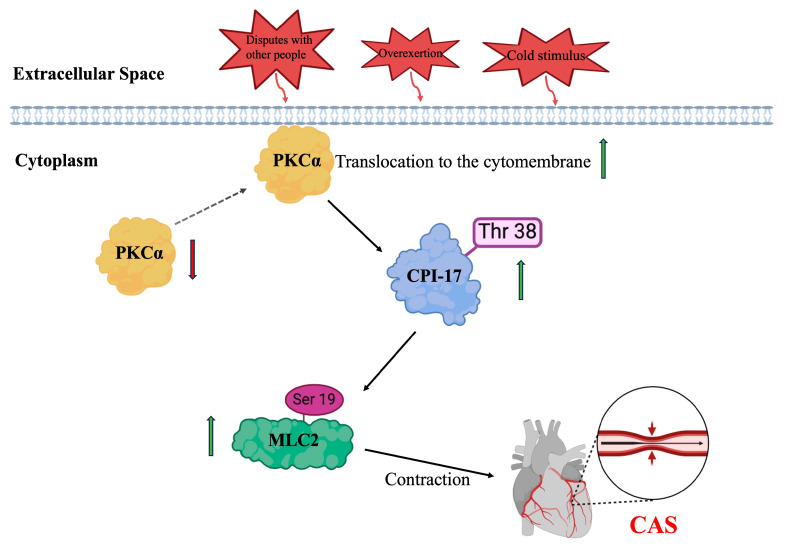
Schematic illustration of PKC activation of the CPI-17/MLC2 pathway to induce CAS. Upon predisposing factors (e.g., disputes with other people, overexertion, cold stimulus), PKCα is activated; it translocates from cytoplasm to the cell membrane to exert its effects. PKC promotes the phosphorylation of CPI-17 at threonine 38, which in turn promotes the phosphorylation of MLC2 at serine 19, thereby facilitating coronary artery contraction and eventually contributing to CAS. Thr 38: threonine 38; Ser 19: serine 19. Red arrow represents the decrease of protein expression level; Green arrows represent the increase of protein expression levels.

PKC regulates downstream signals such as endothelin-1 (ET-1), MAPK, and CPI-17, thus playing an important role in vasoconstriction [14,15,16,17]. It is widely distributed, and its isoforms have species and tissue specificity. The PKC superfamily contains 12 isoenzymes, of which 3 (PKCα, -δ, and -ɛ) are related to Ca^2+^ sensitization and contraction induced by G protein-coupled receptor (GPCR) stimulation in the VSM [33]. PKC is usually present in cells in an inactive state; when activated, it translocates from cytoplasm to the cell membrane to exert its effects. Our results show that PKCα was translocated from cytoplasm to the cell membrane in the rat CAS stimulation model. In our in vitro experiments, we also found that Ang II induced PKCα translocation from the cytoplasm in hCASMCs, which was accompanied by cell contraction. However, Ang II did not induce PKCδ or PKCε redistribution. The above-described results show that PKCα translocation participated in CAS. When signaling molecules act on receptors on the cell membrane, they can activate G proteins and then PLC, which hydrolyzes phosphatidylinositol-4,5-bisphosphate (PIP2) to inositol trisphosphate (IP3) and diacylglycerol (DAG). DAG activates PKC under the synergistic effect of Ca^2+^, thereby regulating downstream signaling [24]. CPI-17 is considered an endogenous mediator of the PKC signaling pathway [23]. We hypothesized that PKC might play an important role in CAS via the CPI-17/MLC2 pathway; to prove that hypothesis, we treated hCASMCs with two PKC-specific inhibitors (GF-109203X and calphostin C) and observed changes in critical proteins in the CPI-17/MLC2 pathway. GF-109203X and calphostin C are two PKC inhibitors with different mechanisms [24]. GF-109203X acts on the ATP-binding site to inhibit the PKC enzyme and therefore inhibits PKC phosphotransferase activity without inhibiting its translocation. Calphostin C competes at the binding site for DAG and phorbol esters and therefore inhibits both PKC phosphotransferase activity and its translocation. However, both inhibitors inhibited PKC signal transduction. Therefore, it is well explained that both GF-109203X and calphostin C could inhibit the Ang II-induced increase in CPI-17 and MLC2 phosphorylation levels. Therefore, our study supports the hypothesis that PKC activates the CPI-17/MLC2 pathway to induce CAS (Figure 9).

Lastly, we evaluated p-CPI-17 and p-MLC2 levels in coronary artery tissues from three deceased patients suspected of having suffered CAS-induced cardiac death. IHC staining results indicated that coronary artery tissues were positive for p–CPI-17 in these cases compared with tissues from three non-cardiac death cases. In addition, coronary artery segments and neighboring interstitial arterioles in suspected CAS-induced cardiac-death cases showed positive p-MLC2 staining. This was consistent with previous studies’ findings that p-MLC2 is a postmortem molecular marker of CAS [6]. Taken together, these results show that p-CPI-17 and p-MLC2 could be used as potential molecular markers for postmortem diagnosis of CAS. 

There are several limitations to this study. First, the number of autopsy cases available to evaluate the validation of candidate biomarkers in this study was small, and validation with a larger sample size would strengthen the conclusion. Second, the postmortem interval of forensic samples used in our study was within 2–5 days. In order to increase the application of the conclusion in forensic practice, verification of forensic samples at different autopsy times should be expanded.

In summary, in this study, we explored the pathological molecular mechanisms of CAS to provide a theoretical basis and reference for identification of diagnostic markers for use in forensic practice to confirm suspected CAS-induced SCD.

## 4. Materials and Methods

### 4.1. Human Subjects and Ethical Statements

We obtained LADA paraffin block specimens from three non-cardiac death cases and three CAS-induced cardiac death cases from the Forensic Identification Center of Hebei Medical University (grant No.: 2023023). Written informed consent was obtained from the relatives of each deceased subject based on HMU Institutional Review Board requirements.

### 4.2. Experimental Animals

Male Sprague–Dawley (SD) rats weighing 250 ± 10 g were purchased from Beijing Vital River Laboratory Animal Technology Co., Ltd. (Beijing, China). All the rats were provided with food and water ad libitum and were kept in a climate-controlled environment, at a consistent temperature (22 ± 2 °C), humidity (60–65%), and 12 h light/dark cycle. The rats were randomly divided into the control group and CAS group (*n* = 5 rats per group). All procedures followed the National Institutes of Health guidelines and were approved by the Institutional Review Board for Animal Experiments at Hebei Medical University.

### 4.3. Animal Treatments and Experimental Procedure

The model of spasm provocation in the rat coronary artery was established as previously described [7]. In brief, the rats were anesthetized with 2% pentobarbital sodium (0.3 mL/100 g, i.p.). The rat model of CAS was constructed by injecting pituitrin (1.7 U/kg) into the tail vein of the rats. The changes in the S-T segment in the rats were monitored as the criteria for determining coronary artery spasm in rats. The electrocardiogram was monitored using a PowerLab system (AD Instruments, Bella Vista, Australia). The rats were fixed on the experimental platform, and the platinum electrodes were inserted subcutaneously by way of a lead II electrode connection. The rat ECGs were subsequently recorded using a bio-amp.

### 4.4. Tissue Preparation

All rats were anesthetized by intraperitoneal injection of 2% pentobarbital sodium. Myocardial and the left anterior descending coronary artery tissues for staining were collected and immediately fixed in 10% formalin. After ethanol gradient dehydration and paraffin embedding, the tissue was cut into 5 μm sections for HE staining, immunohistochemical staining, and immunofluorescence staining. Myocardial and the left anterior descending coronary artery tissues were immediately collected from other rats. Part of the tissue was fixed in 4% glutaraldehyde solution and used for transmission electron microscopy, and the other part was immediately stored at −80 °C and used for Western blotting.

### 4.5. Cells and Cell Culture

hCASMCs (CP-H167, Procell, Wuhan, China) were grown in hCASMCs complete medium (CM-H167, Procell, Wuhan, China) under a water-saturated atmosphere of 95% air and 5% CO_2_ at 37 °C. Cells were determined to be SMCs according to morphology and expression of α-SMA. To establish a cell model of contraction, hCASMCs were subjected to serum-deprived DMEM incubation 30 min prior to exposure to angiotensin II (AngII) (HY-13948, MCE, Newark, NJ, USA) at serial concentrations (0, 0.05, 0.1, 0.5 and 1 μM) or for different time durations (0, 5, 15, 30 and 60 min) at a fixed dose (0.1 μM). In the following experiments, the cultured hCASMCs were randomly assigned to six groups, cultured under different conditions. In the control group, the cells were maintained in normal DMEM. In the AngII group, cells were cultured in culture medium containing 0.1 μM of AngII for 30 min. In the GF-109203X (HY-13867, MCE, Newark, NJ, USA) group, cells were cultured in culture medium containing 5 μM of GF-109203X for 1 h. In the calphostin C (CAS No. 121263-19-2, Cayman, Ann Arbor, MI, USA) group, cells were cultured in culture medium containing 3 μM of calphostin C for 1 h. In the AngII + GF-109203X group, cells were cultured in culture medium containing 5 μM of GF-109203X first, before 0.1 μM of AngII was added and the cells were cultured for 30 min. In the AngII + calphostin C group, cells were cultured in culture medium containing 3 μM of calphostin C first, before 0.1 μM of AngII was added and the cells were cultured for 30 min.

### 4.6. HE Staining

Paraffin sections were dried at 60 °C for 40 min. After dehydration using an ethanol gradient, HE staining was performed according to the manufacturer’s protocols (G1120; Solarbio, Beijing, China) and then observed under a light microscope (Olympus IX71; Olympus Corp., Tokyo, Japan).

### 4.7. Transmission Electron Microscopy (TEM)

The myocardial tissue was fixed with 2.5% glutaraldehyde for 48 h. After overnight fixation, samples were postfixed in 1% osmium tetroxide, dehydrated through graded concentrations of ethanol, infiltrated, and embedded in Epon 812 at 60 °C for 48 h. Ultrathin sections were cut with a diamond knife, mounted on formvar-coated slot grids, and then stained with 2% uranyl acetate and lead citrate for 15 min, respectively. The images were taken with a HT7700 model Hitachi TEM (Hitachi, Tokyo, Japan).

### 4.8. Immunohistochemistry (IHC) Analysis

Paraffin cross-sections (5-μm thick) from coronary arteries and the adjacent heart tissues were deparaffinized with xylene and rehydrated in a graded ethanol series. The cleared sections were preprocessed using a microwave for antigen retrieval, followed by incubation in 3% H_2_O_2_ for 30 min at room temperature. The tissue sections were blocked in goat serum at 37 °C for 40 min and then incubated with the following primary antibodies overnight at 4 °C: anti-p-CPI-17 (1:100, AF3473, Affinity Biosciences, Cincinnati, OH, USA) and anti-p-MLC2 (1:50, 3671, Cell Signaling Technology, Boston, MA, USA). A horseradish peroxidase (HRP)-conjugated goat anti-rabbit secondary antibody was then incubated with the slides at 37 °C for 40 min. Finally, the sections were incubated with diaminobenzidine (DAB) and counterstained with hematoxylin to visualize the nuclei.

### 4.9. Immunofluorescence (IF) Analysis

The cleared sections were preprocessed using a microwave for antigen retrieval before blocking with goat serum at 37 °C for 40 min. The sections were incubated with anti-p-CPI-17 antibody (1:100, AF3473, Affinity Biosciences, Cincinnati, OH, USA) and anti-p-MLC2 antibody (1:50, 3671, Cell Signaling Technology, Boston, MA, USA) at 4 °C overnight. After that, the sections were incubated with the fluorescent secondary antibodies Dylight-647 (1:200, ab150079, Abcam, Cambridge, MA, USA) at 37 °C for 1 h. Finally, DAPI-containing anti-fluorescence quenching medium (0100-20; SouthernBiotech, Birmingham, AL, USA) was used for mounting. A laser confocal microscope (TCS SP8; Leica, Wetzlar, Germany) was used for single-layer scanning of the fluorescent images. ImageJ (version 1.8.0; U.S. National Institutes of Health, Bethesda, MD, USA, https://imagej.net/ij/index.html, accessed on 18 June 2023) was used to quantitate fluorescence intensity.

### 4.10. Immunocytochemistry (ICC) Analysis

hCASMCs cultured on 24-well coverslips were removed from the culture medium. The coverslips were cleaned with 1× phosphate-buffered saline (PBS) twice, and 4% paraformaldehyde was applied for fixation at room temperature for 15 min. Cells were permeabilized in 0.5% Triton-X-100 for 15 min and blocked with normal goat serum for 30 min at room temperature. Primary antibodies against p-CPI-17 (1:100, AF3473, Affinity Biosciences, Cincinnati, OH, USA), p-MLC2 (1:50, 3671, Cell Signaling Technology, Boston, MA, USA), PKCα (1:100, AF6196, Affinity Biosciences, Cincinnati, OH, USA), PKCδ (1:200, ab182126, Abcam, Cambridge, MA, USA), and PKCɛ (1:100, AF7845, Affinity Biosciences, Cincinnati, OH, USA) were added to coverslips at 4 °C overnight. Then, the coverslips were incubated with the fluorescent secondary antibodies Dylight-647 (1:200, ab150079, Abcam, Cambridge, MA, USA) or Dylight-488 (1:200, ab150077, Abcam, Cambridge, MA, USA) at 37 °C for 1 h. Finally, the coverslips were washed with PBS three times before DAPI-containing anti-fluorescence quenching medium (0100-20, SouthernBiotech, Birmingham, AL, USA) was used for mounting. A laser confocal microscope (TCS SP8; Leica, Wetzlar, Germany) was used for single-layer scanning of the fluorescent images. ImageJ (version 1.8.0; U.S. National Institutes of Health, Bethesda, MD, USA, https://imagej.net/ij/index.html, accessed on 18 June 2023) was used for quantitation of fluorescence intensity.

### 4.11. Western Blot Analysis 

Protein levels of p-CPI-17, CPI-17, p-MLC2, MLC2, PKCα, PKCδ, PKCɛ, ATP1α1, and GAPDH were examined via Western blotting. In brief, the left anterior descending coronary artery tissues and hCASMCs were lysed with RIPA buffer (Solarbio, Beijing, China) supplemented with protease inhibitors (RW0102, Report biotech, Shijiazhuang, Hebei, China) and phosphatase inhibitors (RW0103, Report biotech, Shijiazhuang, Hebei, China) to obtain total protein. Lysates were centrifuged at 10,000 rpm/min for 15 min at 4 °C, and the supernatant was collected. The concentrations of protein in the supernatant were measured using a bicinchoninic acid (BCA) kit (PC0020, Solarbio, Beijing, China). Equal amounts of protein samples were subjected to 10% SDS-PAGE (SW155-02, Seven, Beijing, China) or 12% SDS-PAGE (SW156-02, Seven, Beijing, China) and transferred to PVDF membranes. The membranes were blocked with 5% bovine serum albumin (4240 GR, Einhausen, Biofroxx, Germany) in TBST at 37 °C for 1 h and then respectively incubated with primary antibodies against p-CPI-17 (1:1000, AF3473, Affinity Biosciences, Cincinnati, OH, USA), CPI-17 (1:1000, AF6473, Affinity Biosciences, Cincinnati, OH, USA), p-MLC2 (1:1000, 3671, Cell Signaling Technology, Boston, MA, USA), MLC2 (1:1000, DF7911, Affinity Biosciences, Cincinnati, OH, USA), PKCα (1:1000, AF6196, Affinity Biosciences, Cincinnati, OH, USA), PKCδ (1:2000, ab182126; Abcam, Cambridge, MA, USA), PKCɛ (1:1000, AF7845, Affinity Biosciences, Cincinnati, OH, USA), ATP1α1 (1:1000, 3010, Cell Signaling Technology, Boston, MA, USA), and GAPDH (ET1601-4, Huabio, Hangzhou, China) at 4 °C overnight. They were then incubated with fluorophore-conjugated secondary antibody (1:10,000; C50317-02, Rockland, Philadelphia, PA, USA). The emitted light was detected and analyzed using an Odyssey gel imaging system (LI-COR, Lincoln, NE, USA) and relative target band intensity was normalized to Na^+^-K^+^ ATPase or GAPDH on ImageJ.

### 4.12. Plasma Membrane Extraction 

We extracted plasma membrane as previously described [34]. A plasma membrane protein isolation kit (SM-005; Invent Biotechnologies, Plymouth, MN, USA) was used to isolate the plasma membrane. We then placed tissues in centrifuge tubes for homogenization and centrifuged the tubes at 16,000× *g* for 30 s, after which the supernatant was discarded. The pellet (containing cell nuclei) was resuspended and then recentrifuged at 700× *g* for 1 min. We transferred the supernatant to a new centrifuge tube and centrifuged it at 4 °C and 16,000× *g* for 30 min. The supernatant contained cytoplasmic components, and the pellet contained the entire membrane component (including organelles and plasma membrane). The pellet was resuspended and centrifuged at 4 °C and 7800× *g* for 5 min. We collected the supernatant, added 1.6 mL pre-cooled PBS, and inverted the tube a few times. Next, tubes were centrifuged at 16,000× *g* for 30 min. The supernatant was discarded and the pellet (containing plasma membrane) was retained.

### 4.13. Determination of Serum Myocardial-Enzyme Levels

Whole blood was left to stand at 4 °C for 2 h and then centrifuged at 4 °C and 3000 rpm/min for 15 min. We collected the supernatant and measured serum CK, CK-MB, LDH, and LDH1 concentrations using a fully automatic biochemical analyzer (Chemray 240; Rayto Life and Analytical Sciences Co., Ltd., Shenzhen, China).

### 4.14. Statistical Analysis

All data are shown as means ± SEM. A two-tailed Student’s *t*-test was used for comparison of means between groups, while analysis of variance (ANOVA) was used for comparisons of ≥3 groups, followed by a Bonferroni post hoc test. A value of *p* < 0.05 was considered significant. GraphPad Prism (version 9.5.0; GraphPad Software, San Diego, CA, USA, https://www.graphpad.com, accessed on 27th July 2023) was used for statistical analysis and graphing.

## Data Availability

The original contributions presented in this study are included in the article/Supplementary Materials, further inquiries can be directed to the corresponding authors.

## Supplementary Materials

The supporting information can be downloaded at: https://www.mdpi.com/article/10.3390/ijms25052941/s1.

## References

[1] Matta A., Bouisset F., Lhermusier T., Campelo-Parada F., Elbaz M., Carrié D., Roncalli J. (2020). Coronary Artery Spasm: New Insights. J. Interv. Cardiol..

[2] Yasue H., Mizuno Y., Harada E. (2019). Coronary artery spasm—Clinical features, pathogenesis and treatment. Proc. Jpn. Acad. Ser. B Phys. Biol. Sci..

[3] Yasue H. (2022). Coronary artery spasm: Not rare but not fully understood yet. Int. J. Cardiol..

[4] Myerburg R.J., Junttila M.J. (2012). Sudden cardiac death caused by coronary heart disease. Circulation.

[5] Teragawa H., Oshita C., Ueda T. (2018). Coronary spasm: It’s common, but it’s still unsolved. World J. Cardiol..

[6] Li L., Li Y., Lin J., Jiang J., He M., Sun D., Zhao Z., Shen Y., Xue A. (2016). Phosphorylated Myosin Light Chain 2 (p-MLC2) as a Molecular Marker of Antemortem Coronary Artery Spasm. Med. Sci. Monit..

[7] Xue A., Lin J., Que C., Yu Y., Tu C., Chen H., Liu B., Zhao X., Wang T., Ma K. (2018). Aberrant endoplasmic reticulum stress mediates coronary artery spasm through regulating MLCK/MLC2 pathway. Exp. Cell Res..

[8] Balistrieri A., Makino A., Yuan J.X. (2023). Pathophysiology and pathogenic mechanisms of pulmonary hypertension: Role of membrane receptors, ion channels, and Ca(^2+^) signaling. Physiol. Rev..

[9] Ge J., Han T., Li X., Shan L., Zhang J., Hong Y., Xia Y., Wang J., Hou M. (2018). S-adenosyl methionine regulates calcium channels and inhibits uterine smooth muscle contraction in rats with infectious premature delivery through the transient receptor protein 3/protein kinase Cβ/C-kinase-activated protein phosphatase-1 inhibitor of 17 kDa signaling pathway. Exp. Ther. Med..

[10] Morin C., Fortin S., Cantin A.M., Rousseau E. (2011). Docosahexaenoic acid derivative prevents inflammation and hyperreactivity in lung: Implication of PKC-Potentiated inhibitory protein for heterotrimeric myosin light chain phosphatase of 17 kD in asthma. Am. J. Respir. Cell Mol. Biol..

[11] Boopathi E., Gomes C., Zderic S.A., Malkowicz B., Chakrabarti R., Patel D.P., Wein A.J., Chacko S. (2014). Mechanical stretch upregulates proteins involved in Ca^2+^ sensitization in urinary bladder smooth muscle hypertrophy. Am. J. Physiol. Cell Physiol..

[12] Xie Z., Su W., Guo Z., Pang H., Post S.R., Gong M.C. (2006). Up-regulation of CPI-17 phosphorylation in diabetic vasculature and high glucose cultured vascular smooth muscle cells. Cardiovasc. Res..

[13] Liu Z., Khalil R.A. (2018). Evolving mechanisms of vascular smooth muscle contraction highlight key targets in vascular disease. Biochem. Pharmacol..

[14] Kadokami T., Shimokawa H., Fukumoto Y., Ito A., Takayanagi T., Egashira K., Takeshita A. (1996). Coronary artery spasm does not depend on the intracellular calcium store but is substantially mediated by the protein kinase C-mediated pathway in a swine model with interleukin-1 beta in vivo. Circulation.

[15] Allahdadi K.J., Duling L.C., Walker B.R., Kanagy N.L. (2008). Eucapnic intermittent hypoxia augments endothelin-1 vasoconstriction in rats: Role of PKCdelta. Am. J. Physiol. Heart Circ. Physiol..

[16] Martinka P., Lai E.Y., Fähling M., Jankowski V., Jankowski J., Schubert R., Gaestel M., Persson A.E., Persson P.B., Patzak A. (2008). Adenosine increases calcium sensitivity via receptor-independent activation of the p38/MK2 pathway in mesenteric arteries. Acta Physiol..

[17] Sun J., Tao T., Zhao W., Wei L., She F., Wang P., Li Y., Zheng Y., Chen X., Wang W. (2019). CPI-17-mediated contraction of vascular smooth muscle is essential for the development of hypertension in obese mice. J. Genet. Genom..

[18] Svendsen E., Tindall A.R. (1988). The internal elastic membrane and intimal folds in arteries: Important but neglected structures?. Acta Physiol. Scand. Suppl..

[19] Qin X., Hou X., Zhang K., Li Q. (2019). α(1D)-adrenoceptor involves the relaxation effect of farrerol in rat aortic vascular smooth muscle cells. Eur. J. Pharmacol..

[20] Yang Q., Fujii W., Kaji N., Kakuta S., Kada K., Kuwahara M., Tsubone H., Ozaki H., Hori M. (2018). The essential role of phospho-T38 CPI-17 in the maintenance of physiological blood pressure using genetically modified mice. FASEB J..

[21] Dallas A., Khalil R.A. (2003). Ca^2+^ antagonist-insensitive coronary smooth muscle contraction involves activation of epsilon-protein kinase C-dependent pathway. Am. J. Physiol. Cell Physiol..

[22] Woodsome T.P., Eto M., Everett A., Brautigan D.L., Kitazawa T. (2001). Expression of CPI-17 and myosin phosphatase correlates with Ca(^2+^) sensitivity of protein kinase C-induced contraction in rabbit smooth muscle. J. Physiol..

[23] Anjum I. (2018). Calcium sensitization mechanisms in detrusor smooth muscles. J. Basic. Clin. Physiol. Pharmacol..

[24] Ringvold H.C., Khalil R.A. (2017). Protein Kinase C as Regulator of Vascular Smooth Muscle Function and Potential Target in Vascular Disorders. Adv. Pharmacol..

[25] Slavich M., Patel R.S. (2016). Coronary artery spasm: Current knowledge and residual uncertainties. Int. J. Cardiol. Heart Vasc..

[26] Kunadian V., Chieffo A., Camici P.G., Berry C., Escaned J., Maas A., Prescott E., Karam N., Appelman Y., Fraccaro C. (2020). An EAPCI Expert Consensus Document on Ischaemia with Non-Obstructive Coronary Arteries in Collaboration with European Society of Cardiology Working Group on Coronary Pathophysiology & Microcirculation Endorsed by Coronary Vasomotor Disorders International Study Group. Eur. Heart J..

[27] Kaski J.C., Crea F., Gersh B.J., Camici P.G. (2018). Reappraisal of Ischemic Heart Disease. Circulation.

[28] Lin Z., Lin X., Zhao X., Xu C., Yu B., Shen Y., Li L. (2022). Coronary Artery Spasm: Risk Factors, Pathophysiological Mechanisms and Novel Diagnostic Approaches. RCM.

[29] Roberts W.C., Curry R.C., Isner J.M., Waller B.F., McManus B.M., Mariani-Costantini R., Ross A.M. (1982). Sudden death in Prinzmetal’s angina with coronary spasm documented by angiography. Analysis of three necropsy patients. Am. J. Cardiol..

[30] Factor S.M., Cho S. (1985). Smooth muscle contraction bands in the media of coronary arteries: A postmortem marker of antemortem coronary spasm?. J. Am. Coll. Cardiol..

[31] Lanza G.A., Careri G., Crea F. (2011). Mechanisms of coronary artery spasm. Circulation.

[32] Chiba Y., Tanabe M., Sakai H., Kimura S., Misawa M. (2010). A functional interaction between CPI-17 and RACK1 proteins in bronchial smooth muscle cells. Biochem. Biophys. Res. Commun..

[33] Mueed I., Zhang L., MacLeod K.M. (2005). Role of the PKC/CPI-17 pathway in enhanced contractile responses of mesenteric arteries from diabetic rats to alpha-adrenoceptor stimulation. Br. J. Pharmacol..

[34] Yang J., Song X., Chen Y., Lu X.A., Fu Y., Luo Y. (2014). PLCγ1-PKCγ signaling-mediated Hsp90α plasma membrane translocation facilitates tumor metastasis. Traffic.

