# Supplementary material for: Phosphorylated CPI-17 and MLC2 as Biomarkers of Coronary Artery Spasm–Induced Sudden Cardiac Death
Source: Int J Mol Sci. 2024 Mar 3;25(5):2941. doi: 10.3390/ijms25052941 (PMC10932290; doi:10.3390/ijms25052941)
Supplement: Supplementary file 1 [file ijms-25-02941-s001.zip › ijms-2833862-supplementary.pdf]

Supplementary Material

Supplementary Figure

Supplementary Figure S1

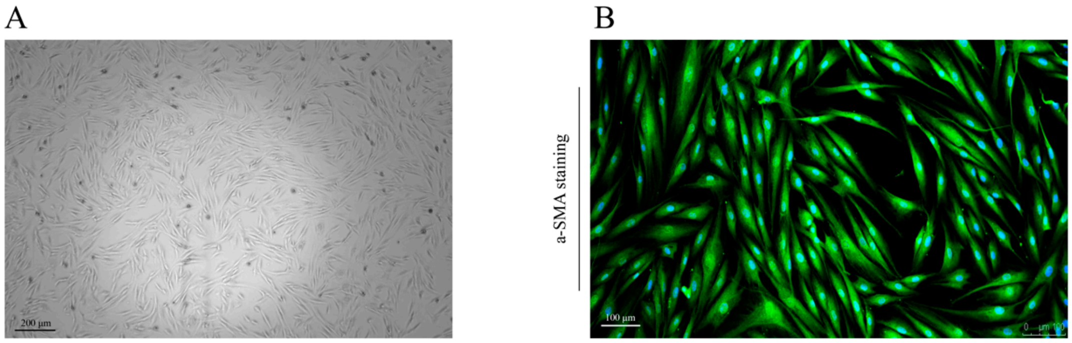

Supplementary Figure S1. Morphological characteristics and identification of hCASMCs. (A) The morphological characteristics of hCASMCs. Scale bar: 200 μm. (B) Expression of a-SMA in hCASMCs was detected by immunofluorescence staining. Scale bar: 100 μm.

Supplementary Figure S2

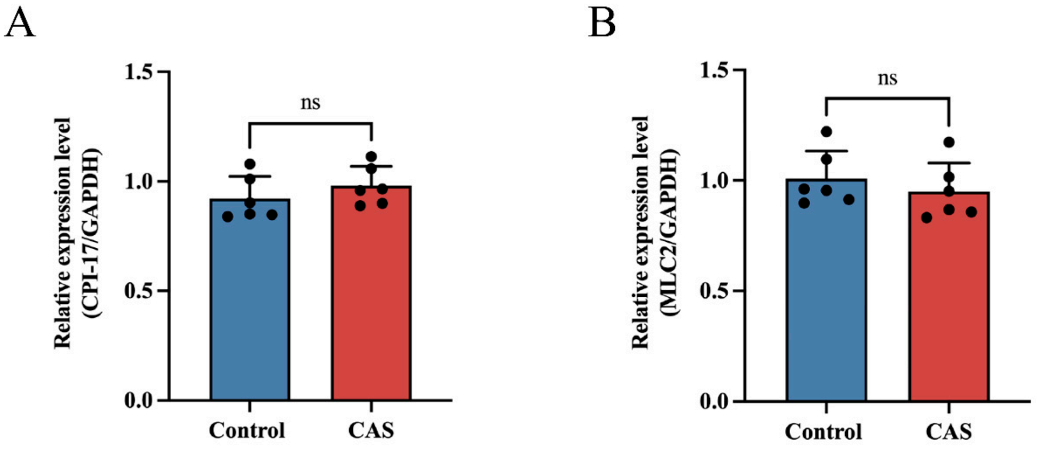

Supplementary Figure S2. The expression of CPI-17 and MLC2 protein levels in CAS rat coronary arteries. (A) Results were expressed as a grayscale ratio of CPI-17 / GAPDH. *n* = 6 per group. (B) Results were expressed as a grayscale ratio of MLC2 / GAPDH. *n* = 6 per group. Data presented as mean ± SEM. ns, no significance.

Supplementary Table

Supplementary Table S1. Autopsy information for collected human left-anterior descending-artery samples.

**Case No.**	**Category**	**Age (y)**	**Gender**	**Circumstances of death**	**PMI, days**	**Gross finding**	**Histological examination**
1	Control	44	M	Hemorrhagic shock due to road traffic accident	2	Mild LADA stenosis	Mild LADA atherosclerosis
2	Control	40	F	Basilar-artery rupture	4	Mild LADA stenosis	Mild LADA atherosclerosis
3	Control	55	M	Fall from height	2	Moderate LADA stenosis	Moderate LADA atherosclerosis
4	CAS	51	M	Quarrel and emotional agitation	2	Mild LADA stenosis	Mild LADA atherosclerosis
5	CAS	48	M	Quarrel and emotional agitation	2	Mild LADA stenosis	Mild LADA atherosclerosis
6	CAS	55	F	Fight resulting in minimal trauma; emotional agitation	5	Moderate LADA stenosis	Moderate LADA atherosclerosis

CAS, coronary-artery spasm; M, male; F, female; PMI, postmortem interval; LADA, left-anterior descending artery.
